# Supplementary material for: Differential activation of programmed cell death in patients with severe SARS-CoV-2 infection
Source: Cell Death Discov. 2023 Nov 20;9:420. doi: 10.1038/s41420-023-01715-4 (PMC10662024; doi:10.1038/s41420-023-01715-4)
Supplement: Supplementary file 1 — Supplementary files [file 41420_2023_1715_MOESM1_ESM.docx]

**SUPPLEMENTARY FILES**

**TITLE**

Differential Activation of Programmed Cell Death in Patients with Severe SARS-CoV-2 Infection

**AUTHORS**

Ashleigh N Riegler^1^, Paul Benson^2^, Kenneth Long^3^, Sixto M Leal Jr^1*^

**SUPPLEMENTARY TABLES AND LEGENDS**

**Supplementary Table S1: Antibodies and Dilutions**

| **Antibody Target** | **Target Pathway** | **Manufacturer** | **Clone** | **Dilution** | **Conjugate** |
| --- | --- | --- | --- | --- | --- |
| **Phosphorylated MLKL** (p Thr357) | Necroptosis | Novus (#954724) | 954724 | 1:200 | *see goat anti-mouse |
| **CD71/Transferrin Receptor 1** | Ferroptosis | Sigma (MABC1765) | 3F3 FMA | 1:1,000 | *see goat anti-mouse |
| **Cleaved Caspase 3** (Asp 175) | Apoptosis | Cell Signaling (9664T) | 5A1E | 1:500 | *see goat anti-rabbit |
| **Cleaved Gasdermin D** (Asp275) | Pyroptosis | Cell Signaling (NC1630035) | E7H9G | 1:500 | *see goat anti-rabbit |
| **Nucleocapsid** protein | SARS-CoV-2 | Abcam (ab281301) | HL5410 | 1:500 | *see goat anti-rabbit |
| Goat **anti-mouse** IgG2a, human adsorbed | N/A | Southern Biotech (OB108007) | *poly | 1:3,000 | Texas Red |
| Goat **anti-Rabbit** IgG(H+L), human adsorbed | N/A | Southern Biotech (OB405030) | *poly | 1:3,000 | AlexaFluor 488 |

**
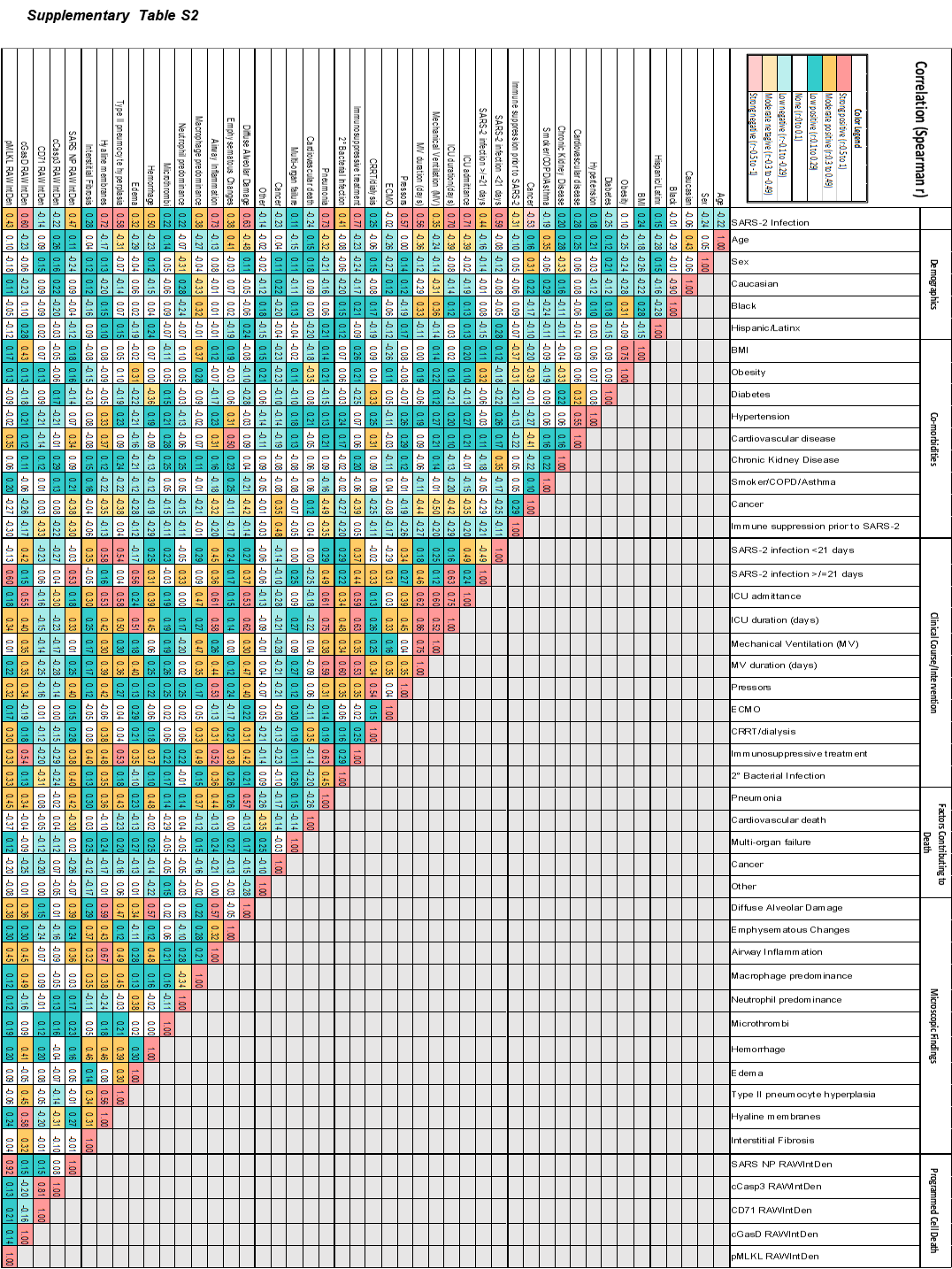
Supplementary Table S2: Correlation between patient characteristics and PCD.** Spearman’s r resulting from correlation between listed characteristics and results. Color shading legend to indicate strength and directionality of correlation

**Supplementary Table S3: Characteristics of patients that died from severe SARS-CoV-2 infection with high versus low viral burden**

|  | **Low SARS-CoV-2 burden (n=19)** | | **High SARS-CoV-2 burden (n=9)** | | ***p-value*** | |
| --- | --- | --- | --- | --- | --- | --- |
|  |  |  |  |  |  |  |
| **Demographics** | | | | |  |  |
| Age (years) | 51.9 | - | 61.2 | - |  | *0.21* |
| Male | 11 | 58% | 6 | 67% |  | *0.65* |
| Female | 8 | 42% | 3 | 33% |  |  |
| Caucasian | 5 | 26% | 4 | 44% |  | *0.34* |
| Black | 12 | 63% | 5 | 56% |  | *0.70* |
| Hispanic/Latinx | 2 | 11% | 0 | 0% |  | *0.31* |
| **Co-morbidities** | | | | |  | |
| BMI | 35.7 | - | 37.7 | - |  | *0.80* |
| Obesity | 14 | 74% | 6 | 67% |  | *0.70* |
| Diabetes | 2 | 11% | 4 | 44% | * | ***0.04*** |
| Hypertension | 10 | 53% | 4 | 44% |  | *0.69* |
| Cardiovascular disease | 10 | 53% | 9 | 100% | * | ***0.01*** |
| Chronic Kidney Disease | 3 | 16% | 4 | 44% |  | *0.10* |
| Smoker/COPD/Asthma | 0 | 0% | 2 | 22% | * | ***0.03*** |
| Cancer | 1 | 5% | 0 | 0% |  | *0.48* |
| Immune suppression prior to SARS-CoV-2 | 1 | 5% | 0 | 0% |  | *0.48* |
| **Clinical Course/ Intervention** | | | | | | |
| SARS-CoV-2 infection < 21 days | 13 | 68% | 3 | 33% | ~ | *0.08* |
| SARS-CoV-2 infection >/= 21 days | 6 | 32% | 6 | 67% |  |  |
| ICU admittance | 19 | 100% | 8 | 89% |  | *0.14* |
| ICU duration (days) | 23.0 | - | 19 | - |  | *0.59* |
| Mechanical Ventilation (MV) | 17 | 89% | 7 | 78% |  | *0.41* |
| MV duration (days) | 20.0 | - | 16.9 | - |  | *0.68* |
| Pressors | 14 | 74% | 9 | 100% |  | *0.09* |
| ECMO | 4 | 21% | 2 | 22% |  | *0.94* |
| CRRT/ dialysis | 8 | 42% | 6 | 67% |  | *0.22* |
| Immune suppressants | 17 | 89% | 9 | 100% |  | *0.31* |
| 2° bacterial lung infection’ | 6 | 32% | 5 | 56% |  | *0.23* |
| **Factors Contributing to Death** | | | | | | |
| Pneumonia | 14 | 74% | 8 | 89% |  | *0.36* |
| Cardiovascular death | 8 | 42% | 2 | 22% |  | *0.31* |
| Multi-organ failure | 1 | 5% | 0 | 0% |  | *0.48* |
| Cancer | 0 | 0% | 0 | 0% |  | *-* |
| Other` | 5 | 26% | 2 | 22% |  | *0.82* |
| **Vaccination Status** | | | | | | |
| 1. Unvaccinated^ | 16 | 84% | 9 | 100% |  | *0.96* |
| Unknown Status | 3 | 16% | 0 | 0% |  |  |

*-ECMO- extracorporeal membrane oxygenation; CRRT- continuous renal replacement therapy*

*#ANOVA with Fisher's Least Significant Difference (LSD) test was used for continuous variables;Chi Square test was used for qualitative variables*

*’ 2° bacterial lung infection include 45.5% Gram negative bacteria (P.aeruginosa, S. maltophilia, K. pneumoniae, K. aerugenes, P. agglomerans, Achromobacter, Acinetobacter, and Enterobacter, ), 27.3% Gram positive pathogens (methicillin-sensitive or methicillin-resistant S. aureus), and 27.3% mixed infection with both Gram negative and Gram positive bacteria (MRSA with K. pneumoniae, MRSA with P. agglomerans, and S. aureus with E. cloaceae).*

*` Other factors contributing to death include: blunt force trauma, renal failure, cirrhosis, and sepsis*

*^Individuals that died prior to the availability of SARS-CoV-2 vaccines were considered unvaccinated*

**Supplementary Table S4: Microscopic findings in the lungs of patients that died from severe SARS-CoV-2 infection with high versus low viral burden**

|  | **Low SARS-CoV-2 burden (n=19)** | | | **High SARS-CoV-2 burden (n=9)** | | | ***p-value (Scores)*** | | ***p-value***  ***(# cases)*** | |
| --- | --- | --- | --- | --- | --- | --- | --- | --- | --- | --- |
|  | **Cases** | **%** | **Score^** | **Cases** | **%** | **Score^** |  |  |  |  |
|  |  |  |  |  |  |  |  |  |  |  |
| Diffuse alveolar damage | 13 | 68% | - | 6 | 67% | - | - | |  | *0.9260* |
| Emphysematous Changes | 9 | 47% | 1.18 | 6 | 67% | 2.06 |  | *0.29* |  | *0.34* |
| Airway inflammation | 19 | 100% | 2.87 | 9 | 100% | 3.00 |  | *0.88* | ***-*** | |
| *Macrophage-predominant^* | 12 | 63% | - | 6 | 67% | - | - | |  | *0.18* |
| *Neutrophil-predominant^* | 2 | 11% | - | 2 | 22% | - | - | |  | *0.41* |
| *Both^* | 5 | 26% | - | 1 | 11% | - | - | |  | *0.36* |
| Microthrombi | 1 | 5% | - | 3 | 33% | - | - | | ******* | ***0.047*** |
| Hemorrhage | 14 | 74% | 2.29 | 4 | 44% | 1.39 |  | *0.28* |  | *0.1315* |
| Edema | 11 | 58% | 1.89 | 4 | 44% | 1.06 |  | *0.31* |  | *0.51* |
| Type II pneumocyte hyperplasia | 18 | 95% | 3.13 | 4 | 44% | 3.44 |  | *0.71* | ******** | ***0.0025*** |
| Hyaline membranes | 17 | 89% | 2.61 | 7 | 78% | 2.06 |  | *0.51* |  | *0.41* |
| Interstitial fibrosis | 10 | 53% | 1.50 | 4 | 44% | 1.33 |  | *0.84* |  | *0.40* |

*^Severity was scored from H&E stained tissue sections on a scale from 0 (trace) to 4 (most severe).*

**SUPPLEMENTARY FIGURE AND LEGEND**

**
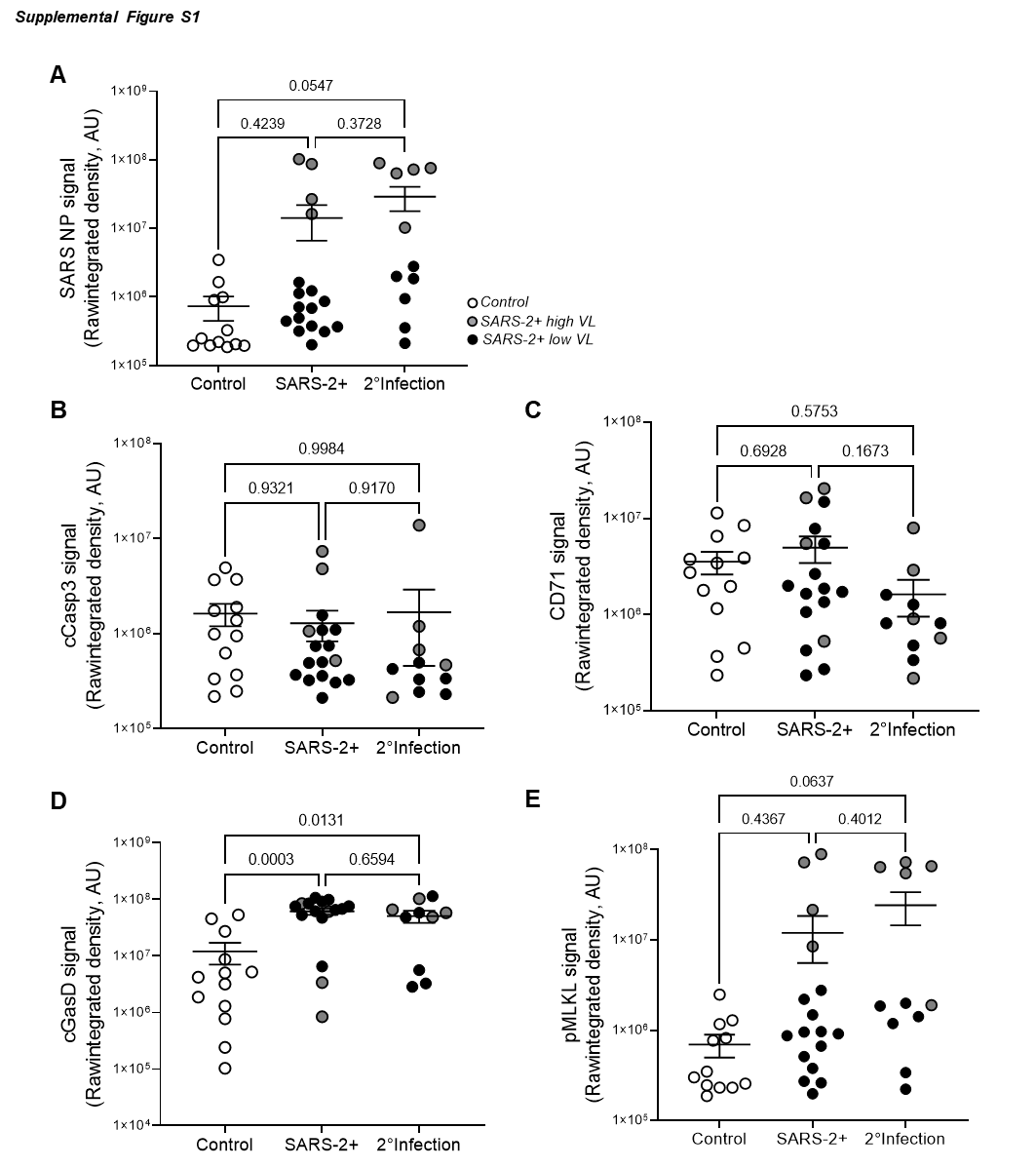
**

**Supplementary Figure S1. Patients with SARS-CoV-2 infection and 2° bacterial pneumonia exhibit no change in the lung programmed cell death profile relative to SARS-CoV-2 only infection.** Immunofluorescence staining was performed on FFPE lung tissues from patients that died from severe SARS-CoV-2 infection with (n= 11) or without (n=17) evidence of 2° bacterial respiratory tract infection. Quantification of raw integrated intensity shows no statistically significant difference in the following parameters: **A.** SARS-CoV-2 nucleoprotein (N protein), **B.** cleaved caspase-3 (cCasp3), **C.** transferrin receptor (CD71), **D.** cleaved gasdermin D (cGasD), and **E.** phosphorylated MLKL (pMLKL).
